# Multifactor analysis and dominant factor identification for coal seam gas drainage borehole instability

**DOI:** 10.1038/s41598-025-32455-3

**Published:** 2025-12-24

**Authors:** Wenyuan Wang, Wei Yang, Zhichao Zhang, Wei Shi

**Affiliations:** 1 School of Safety Engineering, China University of Mining and Technology, Xuzhou, 221116 Jiangsu China; 2 Key Laboratory of Theory and Technology On Coal and Rock Dynamic Disaster Prevention and Control, National Mine Safety Administration, China University of Mining and Technology, Xuzhou, 221116 China

**Keywords:** Gas drainage, Borehole stability, Thermo-hydro-mechanical coupling, Screen pipe, Energy science and technology, Engineering, Solid Earth sciences

## Abstract

To reveal the main controlling factors of instability in gas drainage boreholes in coal seams, this study investigated the main controlling factors of instability in coal seam gas drainage boreholes through Thermo-hydro-mechanical coupled triaxial tests, acoustic emission monitoring, and FLAC 3D simulations. Experimental results demonstrate: (1) Increasing confining pressure significantly enhances coal sample parameters including peak strength (76.1% increase), peak strain (74.4% increase), and elastic modulus (28.1% improvement), while elevated gas pressure reduces peak strength by 22.6% and weakens elastic modulus by 32.0%. Temperature rise induces 37.1% and 42.4% reductions in peak strength and strain respectively. (2) Acoustic emission energy evolution exhibits a "slow-accelerated" two-stage characteristic, with initial energy mutation point stress increasing with confining pressure (36.8–52.8 MPa) but decreasing with gas pressure and temperature elevation. Numerical simulations indicate PP screen pipe support effectively restrains borehole surrounding rock deformation (84.5% vertical displacement reduction) and optimizes stress distribution (35.1% peak stress position reduction), with its synergistic bearing effect increasing coal mass peak stress by 27.19%. Response surface models confirm confining pressure exerts the most significant impact on stability (P < 0.0001), followed by gas pressure and temperature, providing theoretical and technical support for preventing instability in deep coal seam gas extraction boreholes.

## Introduction

The development and utilization of coalbed methane (CBM) resources play a crucial role in diversifying China’s mineral resource utilization and achieving its "dual-carbon" goals^1–3^. Statistical data indicate that approximately 50% of coal mines in China are classified as high-gas or gas-outburst mines^4–6^. As coal mining progresses to greater depths, the geological environments hosting coal seams become increasingly complex, heightening the risks of disasters such as coal and gas outbursts and gas explosions^7^. Consequently, coal seam gas pre-drainage is widely implemented as a regional control measure^8^. However, coal seams are characterized by low strength, high friability, and well-developed fractures^9^, leading to frequent borehole deformation and instability during drilling—manifested as borehole wall collapse, necking, and drill pipe sticking—which severely hinders CBM development^10^. Consequently, in-depth research on borehole instability mechanisms and improved gas drainage efficiency remains urgent challenges.

The application of high-power drilling and extraction equipment, such as kilometer-level drilling rigs and high-vacuum water-ring vacuum pumps, has significantly enhanced borehole lengths and negative pressure at borehole mouths^11,12^, creating favorable conditions for improved gas drainage efficiency. Meanwhile, large-diameter drilling technology has emerged as one of the most direct and effective methods for boosting gas extraction^13–15^. While this technology increases the coal seam contact area, enhances gas desorption efficiency, and expands gas drainage radius, enlarged borehole diameters intensify stress concentrations around the borehole, triggering instability phenomena like collapse and deformation, thereby reducing borehole completion rates and compromising drainage performance. Although the development and application of screened pipe borehole stabilization technologies in soft coal seams have partially improved borehole integrity and effective drainage lengths^16,17^, their practical outcomes in many mines have fallen short of expectations. The multifactorial nature of borehole instability—coupled with limited systematic understanding of multi-field coupling mechanisms—has led to significant variability in the effectiveness of advanced technologies and equipment.

Current research on borehole wall instability in gas drainage has achieved notable progress in theoretical modeling, numerical simulations, and experimental studies. Some scholars have established wellbore stability criteria based on stress equilibrium equations using elastoplastic theory and numerical simulation methods^18–20^, deriving collapse pressure calculations that consider in-situ stress, pore pressure, and strength parameters^4,5^. Other researchers have proposed analytical solutions for plastic zone radius around boreholes, revealing the correlation between plastic zone development and borehole instability^21,22^. Additional studies have employed physical experiments to elucidate the evolution patterns of coal mass instability^23,24^, utilizing CT scanning technology to achieve three-dimensional visualization characterization of fracture networks surrounding boreholes^25–27^. However, further research is still required to investigate the dominant controlling factors of borehole deformation and instability under complex geological conditions.

Particularly, existing studies on gas drainage boreholes often neglect the impact of geothermal temperature^28^. As mining depths increase, rising formation temperatures make thermal stress a crucial factor affecting gas flow in coal seams^29^, leading to thermal expansion-induced fracturing, accelerated thermal motion of gas molecules, and reduced coal strength^30–32^. However, most laboratory experiments overlook in-situ temperature–pressure coupling effects. Consequently, traditional research approaches are inadequate for analyzing gas extraction in deep coal seams, necessitating the incorporation of specific reservoir environmental conditions^33^. Coal stability results from the dynamic equilibrium of multi-field coupling effects involving confining pressure, gas pressure, and temperature^34–36^, requiring more comprehensive consideration of stress and temperature fields in analyzing borehole instability^30,31,35,36^.

Furthermore, significant research gaps exist regarding the stabilizing effects of screen pipes. A nonlinear relationship exists between screen pipe stiffness and surrounding rock deformation^37,38^, with screen pipe-supported boreholes exhibiting approximately 40% greater deformation resistance than conventional open holes^16^. While existing studies have analyzed the supporting capacity of engineering plastics like PVC, PP, and PE^4,5,39^, they fail to account for the dynamic interaction between support structures and coal-rock mass under thermo-hydro-mechanical coupling conditions. These research gaps highlight the need to compare the supporting efficiency of different screen pipe materials under complex conditions and elucidate the energy transfer mechanism between support systems and surrounding rock.

Based on these considerations, this study investigates borehole stability by examining the deformation instability mechanisms and acoustic emission energy damage characteristics of gas-bearing coal under thermo-hydro-mechanical coupling conditions. Using FLAC 3D software, we simulate the instability process of coal seam drainage boreholes to analyze screen pipe effects and optimize material selection. Additionally, we study the mechanical properties of screened coal under axial dynamic loading. Finally, employing response surface methodology, we develop a multifactorial regression model incorporating confining pressure, gas pressure, temperature, support materials, and peak stress to identify dominant instability factors. The findings hold significant practical implications for addressing borehole instability in deep coal seams, improving gas extraction efficiency, and ensuring mine safety and sustainable development.

## Experimental samples and methods

### Experimental samples

**Fig. 1:**
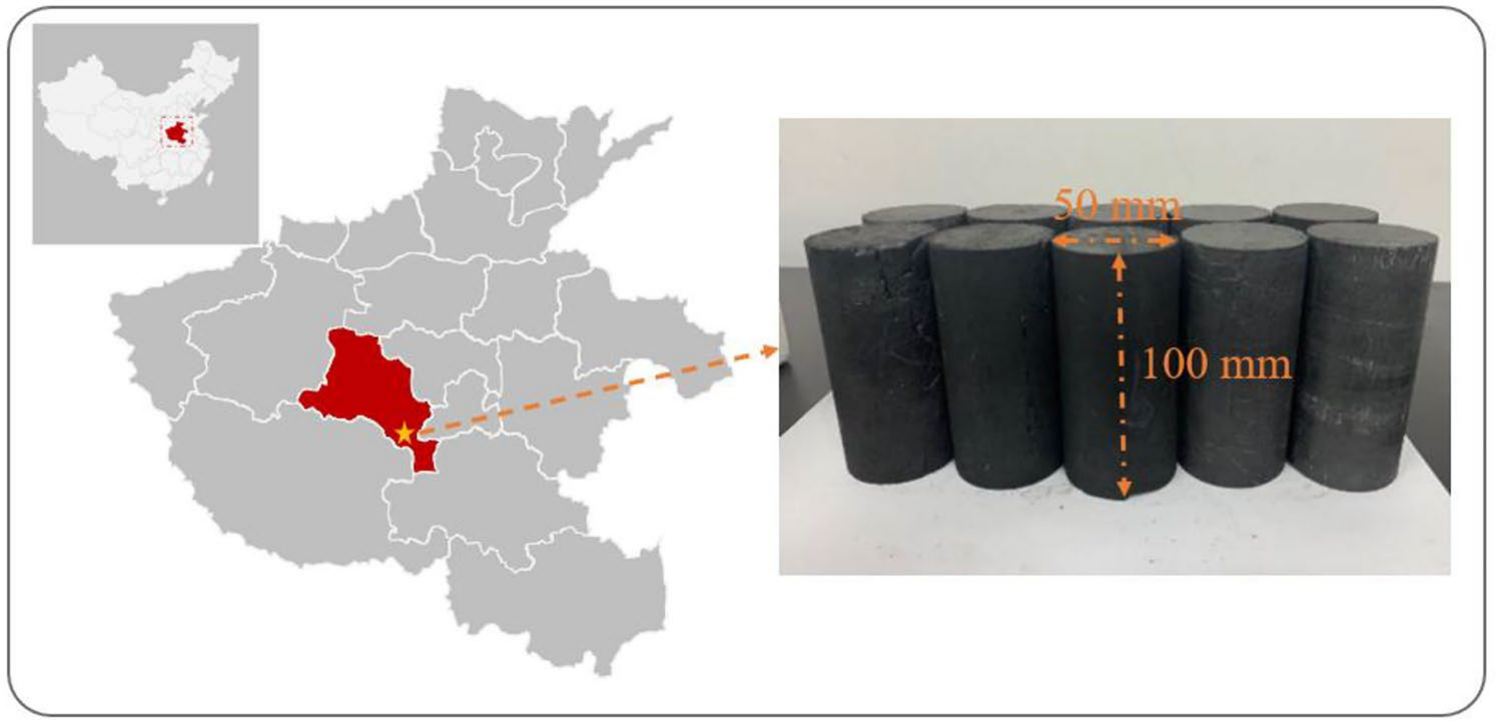
Coal sample preparation.

Fresh coal samples were collected from the J_15_-21030 working face of Pingmei Group’s No.8 Mine and transported to the processing laboratory under sealed conditions. According to experimental requirements, the samples were processed into standard cylindrical specimens measuring φ50 × 100 mm. During machining, special attention was paid to cutting all samples along the same bedding plane direction to minimize potential influencing factors in subsequent experiments. The final processed coal specimens are shown in Fig. 1, with their proximate analysis results presented in Table 1.

**Table 1:** Proximate analysis results of coal samples.

Sampling location	Adsorption constants		Moisture (%)	Ash (%)	Volatiles (%)	True density (g/cm^3^)	Apparent density (g/cm^3^)
	A (m^3^/t)	b (MPa^−1^)					
J_15_-21,030 working face	26.588	0.546	1.67	7.46	33.42	1.41	1.34

### Thermo-hydro-mechanical coupling experiment

**Fig. 2:**
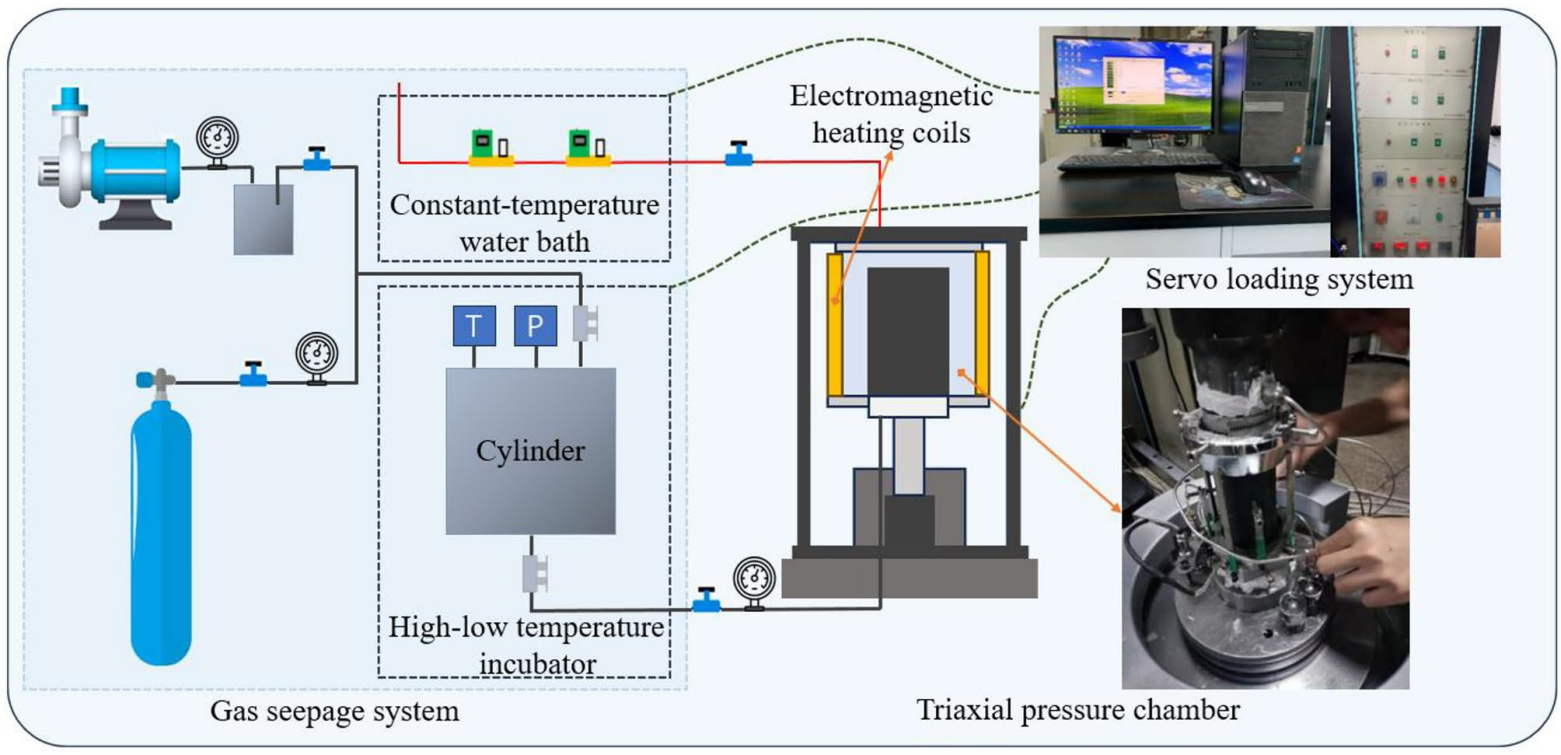
Schematic diagram of the thermo-hydro-mechanical coupling experiment system.

Figure 2 presents the schematic diagram of the experimental system, which primarily consists of four key components: a triaxial pressure chamber, a gas seepage system, a servo loading system, and a temperature control system. The temperature control system incorporates multiple precision elements including a high-low temperature incubator, electromagnetic heating coils, a constant-temperature water bath, and thermal insulation materials surrounding gas pipelines. This comprehensive setup enables precise temperature regulation throughout the entire experimental apparatus, ensuring thermal consistency between gases in the flowmeter and those within both the gas seepage system and triaxial pressure chamber, thereby effectively eliminating experimental errors induced by temperature variations.

The experimental procedure was systematically conducted under controlled conditions as follows, with critical operational parameters detailed in Table 2 (Q-2 group serving as the control group throughout the investigation):

**Table 2:** Experimental scheme diagram.

Coal sample number	Quality (g)	Initial axial pressure (MPa)	Confining pressure (MPa)	Gas pressure (MPa)	Temperature (℃)
Q-1	259.25	12	6	1.4	25
Q-2	260.21	12	8	1.4	25
Q-3	257.99	12	10	1.4	25
Q-4	257.84	12	12	1.4	25
Q-5	255.93	12	8	1.0	25
Q-6	260.79	12	8	1.8	25
Q-7	263.50	12	8	2.2	25
Q-8	260.75	12	8	1.4	35
Q-9	260.22	12	8	1.4	45
Q-10	259.45	12	8	1.4	55

1. *Specimen preparation* Coal samples were loaded into a triaxial compression chamber. 704 silicone sealant was applied to both upper/lower loading platens and sample peripheries. Specimens were encapsulated with heat-shrink tubing and desiccated for > 10 h. The chamber was hermetically sealed, oil-filled, and connected to both the seepage apparatus and vacuum system.
2. *Stress initialization & gas adsorption* Confining pressure was incrementally elevated to 8 MPa, followed by axial stress loading to 12 MPa. The system was thermally equilibrated at 25°C (minimum 2 h stabilization post-temperature attainment). Gas injection at 1.4 MPa pressure persisted for > 12 h until pressure gauge readings from reference the cylinder stabilized. Subsequent outlet valve activation enabled steady-state gas flow, with gas flux data automatically logged at 6-s intervals via computer-controlled acquisition software.
3. *Progressive loading & monitoring* Axial displacement-controlled loading (constant strain rate) was implemented until specimen failure, synchronized with continuous acoustic emission monitoring throughout the deformation process.
4. *Confining pressure variation* Systematically modifying confinement to 6, 10, and 12 MPa while maintaining other parameters constant, the full experimental sequence (Steps 1–3) was replicated to elucidate confining pressure effects on coal deformation mechanics.
5. *Gas pressure modulation* Through sequential adjustment of gas pressure to 1.0, 1.8, and 2.2 MPa under fixed confinement, the experimental protocol (Steps 1–3) was repeated to characterize gas pressure-dependent deformation behavior.
6. *Thermal effect analysis* By regulating all thermal control units to 35, 45, and 55°C respectively, the standard procedure (Steps 1–3) was executed to quantify temperature influence on coal’s deformation instability characteristics.

### Acoustic emission monitoring

**Fig. 3:**
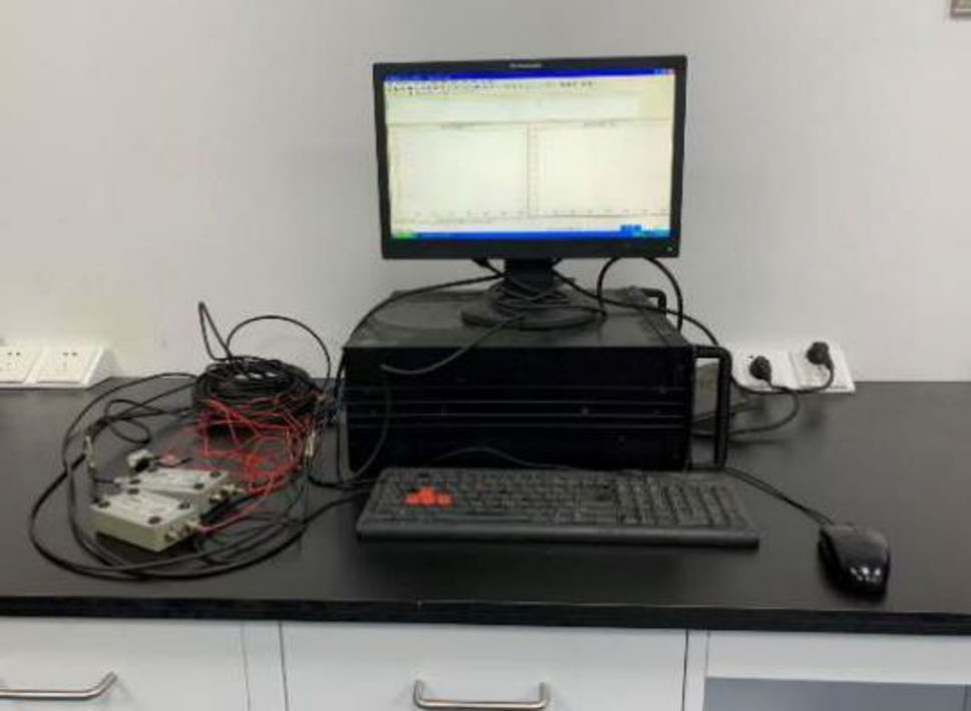
Acoustic emission experimental system.

Figure 3 shows the acoustic emission (AE) experimental system produced by Beijing Kehai Hengsheng Technology Co., Ltd. This system comprises three key components: AE sensors, a preamplifier filter, and a signal processing unit. The AE testing enables non-destructive characterization of fracture development characteristics within coal samples, with energy fluctuations and other parametric features quantitatively reflecting the evolution of internal fractures and pores. By synchronizing AE monitoring with coupled thermo-hydro-mechanical experiments, we achieve comprehensive tracking of energy-based damage evolution throughout the testing process. During triaxial compression tests, the pressure chamber filled with hydraulic oil provides optimal acoustic/mechanical wave propagation conditions, where the short transmission distance renders signal attenuation and time delay negligible for experimental purposes.

## Experimental results and analysis

### Thermo-hydro-mechanical coupling experiment results and analysis

**Fig. 4:**
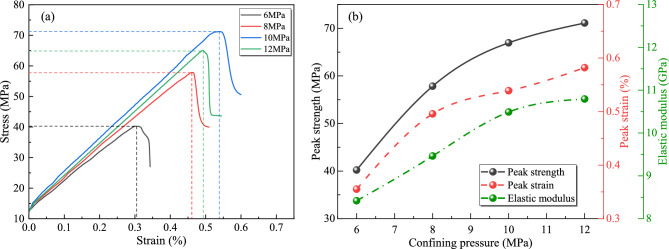
Thermo-hydro-mechanical coupling experimental results under varying confining pressures: (**a**) stress strain curves; (**b**) mechanical characteristic parameters.

The stress–strain curves of triaxial compression tests under varying confining pressures are presented in Fig. 4a. Mechanical characteristic parameters derived from these curves—peak strength, peak strain, and elastic modulus—are statistically summarized in Fig. 4b.

As shown in Fig. 4, the mechanical properties of gas-bearing coal samples exhibit significant enhancement with increasing confining pressure: peak strength rises from 40.22 MPa to 70.85 MPa (76.1% growth), Peak strain expands from 0.31% to 0.53% (74.4% growth), elastic modulus increases from 8.42 GPa to 10.79 GPa (28.1% stiffness improvement). The analysis reveals that the three-dimensional confinement effect induces by elevated confining pressure effectively suppresses the propagation of internal micro-fractures in coal samples while promoting frictional interlocking of closed fractures, thereby enhancing shear strength parameters. Additionally, the compaction-driven densification resulting from confining pressure increases optimizes the contact area and load-bearing pathways of the coal-rock matrix, significantly improving structural rigidity. This confining pressure reinforcement mechanism not only amplifies load-bearing capacity (evidenced by peak strength enhancement) but also suppresses brittle failure modes, facilitating greater plastic deformation (peak strain growth). Concurrently, it strengthens stress transfer efficiency during the elastic phase (elastic modulus elevation). Thus, increased confining pressure substantially fortifies the mechanical integrity of coal samples.

**Fig. 5:**
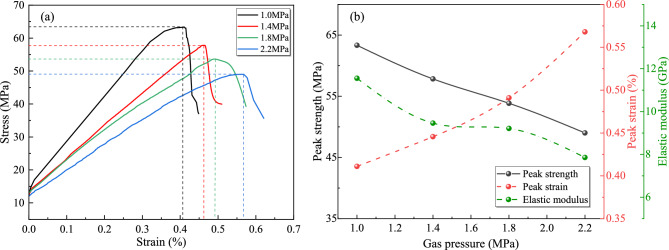
Thermo-hydro-mechanical coupling experimental results under varying gas pressures: (**a**) stress strain curves; (**b**) mechanical characteristic parameters.

The stress–strain curves of triaxial compression tests under varying gas pressures are shown in Fig. 5a. Statistical results of mechanical characteristic parameters are summarized in Fig. 5b.

As illustrated in Fig. 5, the mechanical properties of gas-bearing coal samples undergo significant changes with increasing gas pressure: peak strength decreases from 63.33 MPa to 49.01 MPa (22.6% reduction), peak strain increases from 0.41% to 0.57% (38.2% growth), elastic modulus declines from 11.55 GPa to 7.85 GPa (32.0% stiffness loss). The analysis reveals that free-state gas pressure within fractures reduces the effective confining pressure, thereby weakening the shear strength parameters of the coal-rock matrix. Additionally, the wedging effect induced by gas permeation promotes the propagation of pre-existing fractures and the nucleation of new microcracks, accelerating damage accumulation and degradation of load-bearing structures. The synergistic interaction of these mechanisms manifests as a stiffness reduction (elastic modulus decline) and a transition from brittle to plastic failure modes (peak strain increase). Consequently, gas pressure exerts a strong coupled damaging effect on the mechanical behavior of coal, highlighting its critical role in destabilizing coal mass under gas-rich conditions.

**Fig. 6:**
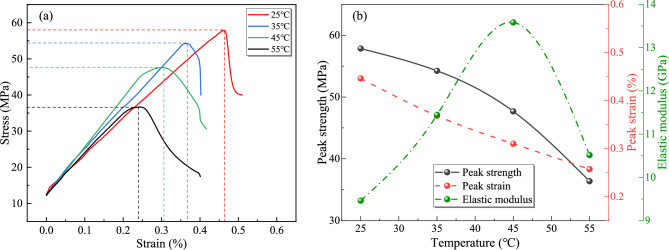
Thermo-hydro-mechanical coupling experimental results under varying temperatures: (**a**) stress strain curves; (**b**) mechanical characteristic parameters.

Figure 6a presents the stress–strain curves of triaxial compression tests under varying temperatures, with statistical results of mechanical characteristic parameters summarized in Fig. 6b.

As illustrated in Fig. 6, the mechanical response of gas-bearing coal samples exhibits significant thermal degradation with increasing temperature: peak strength progressively declines from 57.84 MPa to 36.37 MPa (37.1% reduction), peak strain contracts from 0.45% to 0.26% (42.4% decrease), elastic modulus follows a non-monotonic trend—initially rising from 9.46 GPa to 13.58 GPa before dropping to 10.51 GPa (net increase of 11.1%). The analysis reveals that elevated temperatures disrupt the adsorption equilibrium between coal and gas, increasing desorbed gas volume and pore pressure. This reduces effective principal stress and weakens the load-bearing capacity of the coal matrix. Concurrently, intensified thermal motion of free-state gas molecules generates microscale gas wedging effects, accelerating the propagation and coalescence of pre-existing fractures, thereby accumulating structural damage. At the same time, thermal expansion of the coal matrix drives microcrack extension, reducing coal compactness and synchronously degrading compressive strength and deformation resistance. When temperature exceeds the critical threshold, the synergistic interplay of thermal, stress, and seepage fields reconfigures mesostructural architecture, thereby altering the response pathways of macroscopic mechanical parameters and inducing non-monotonic evolution of properties such as elastic modulus.

### Acoustic emission monitoring results and analysis

**Fig. 7:**
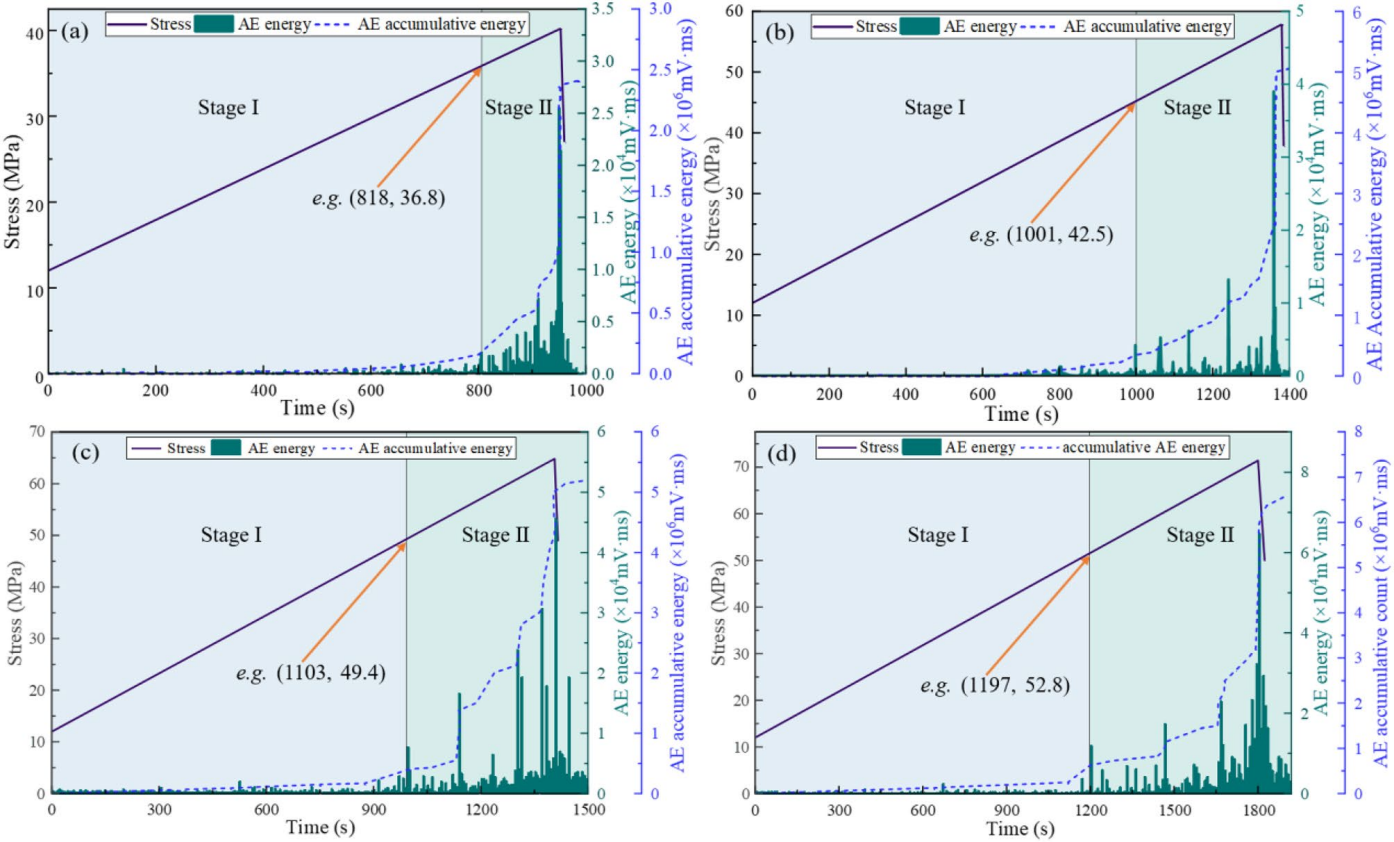
Curves of stress, AE energy, and AE cumulative energy under varying confining pressures: (**a**) 6 MPa; (**b**) 8 MPa; (**c**) 10 MPa; (**d**) 12 MPa.

The physical instability of coal is intrinsically linked to energy dissipation processes, which can be characterized through AE energy analysis^40^. Figure 7 illustrates the relationship between stress, AE energy, and AE cumulative energy under varying confining pressures. The results demonstrate a consistent trend in AE energy evolution across varying confining pressures: AE energy exhibits a fluctuating growth trend with increasing stress, followed by a rapid decline after peak stress. AE cumulative energy demonstrates continuous growth, which can be divided into two distinct stages based on the timing of the first energy surge^14,15,41^:

1. *Slow-growth stage* From data collection initiation to the first energy leap point, the coal undergoes gradual damage accumulation. During this phase, Microcracks develop intermittently, generating sporadic low-frequency AE signals. AE cumulative energy increases at a subdued rate, reflecting progressive but non-critical structural degradation.
2. *Accelerated-growth stage* Spanning from the first energy leap point to experiment termination, this stage features: AE signals intensify in both energy and frequency, with recurrent high-frequency bursts; AE Cumulative energy surges abruptly, accompanied by alternating large- and small-magnitude AE events. This phenomenon indicates that the damage destruction of the coal sample is not a continuous process, but a process of microcrack development, extension, penetration and gradual formation of macroscopic fracture^42^.

Under confining pressures of 6, 8, 10, and 12 MPa, the slow-growth stage persisted for 818 s, 1001 s, 1103 s, and 1197 s respectively. The stress levels at the first energy leap point corresponded to 36.8 MPa, 42.5 MPa, 49.4 MPa, and 52.8 MPa respectively, demonstrating a positive correlation with increasing confining pressure. This indicates that under higher confining pressures, the stress threshold required for initiating brittle micro-damage in coal samples becomes more demanding, reflecting enhanced resistance to instability deformation in the coal mass.

**Fig. 8:**
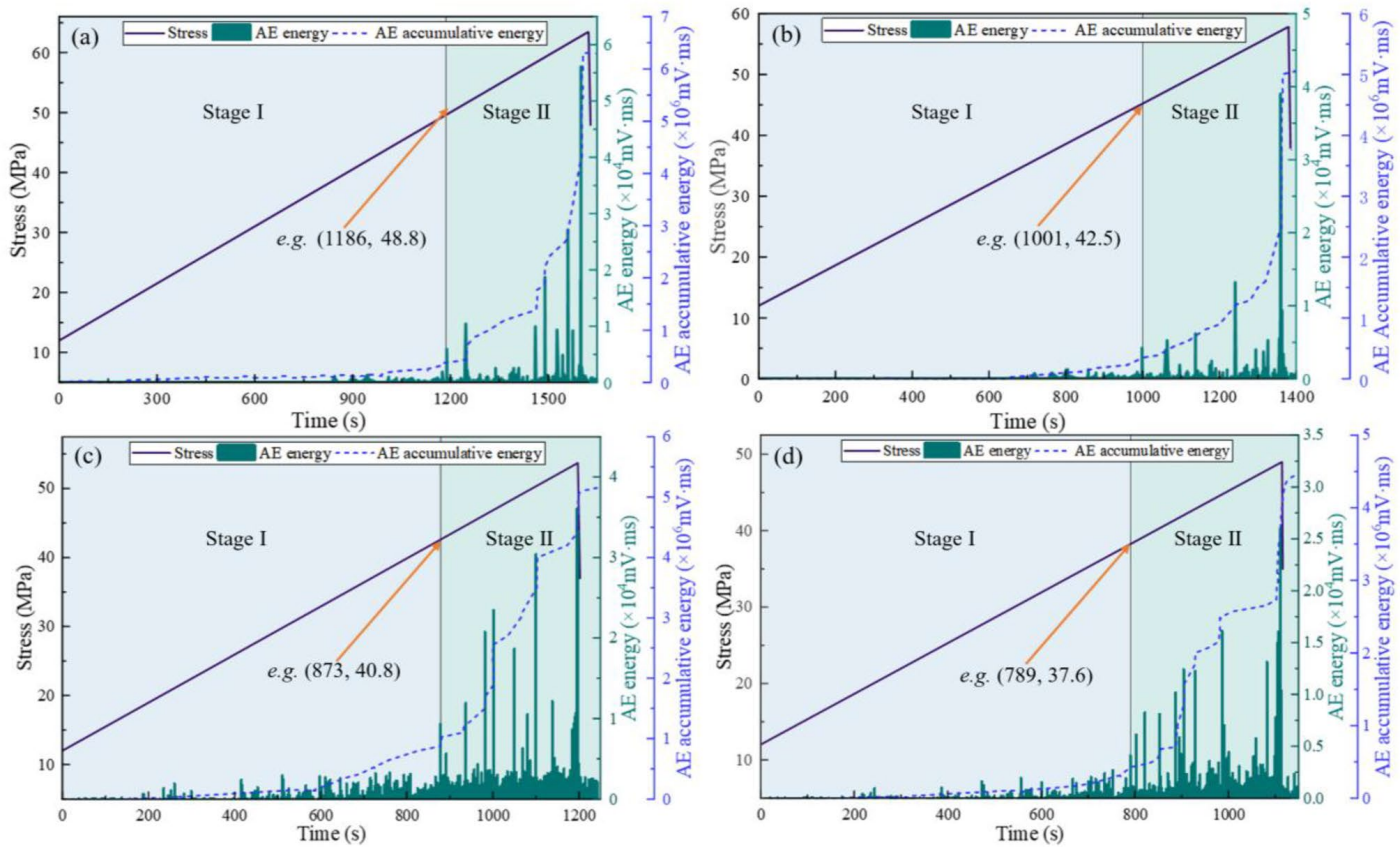
Curves of stress, AE energy, and AE cumulative energy under varying gas pressures: (**a**) 1.0 MPa; (**b**) 1.4 MPa; (**c**) 1.8 MPa; (**d**) 2.2 MPa.

The experimental results of stress-AE energy-AE cumulative energy under varying gas pressures are presented in Fig. 8. At gas pressures of 1.0 MPa, 1.4 MPa, 1.8 MPa, and 2.2 MPa, the slow-growth stage lasted 1186 s, 1001 s, 873 s, and 789 s respectively. The corresponding stresses at the first energy leap point were 48.8 MPa, 42.5 MPa, 40.8 MPa, and 37.6 MPa respectively, showing a decreasing trend with increasing gas pressure. The primary mechanism for this phenomenon lies in the gas existing within the pores and fractures of coal samples. The gas pressure reduces the effective stress by counteracting the external load, thereby decreasing the actual force acting on the coal matrix. Consequently, in the AE monitoring results, the stress corresponding to the first energy transition point decreases with increasing gas pressure.

**Fig. 9:**
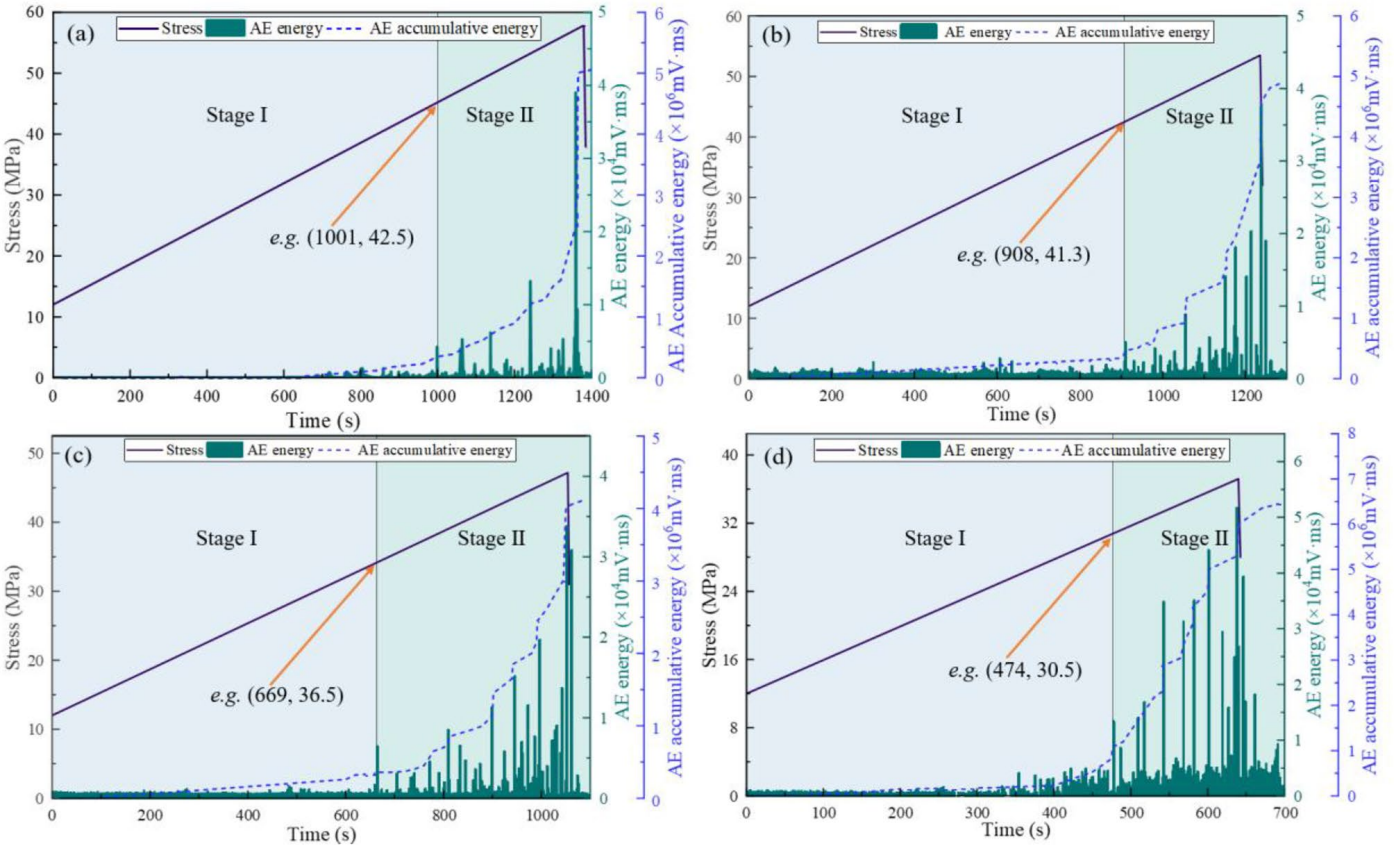
Curves of stress, AE energy, and AE cumulative energy under varying temperatures: (**a**) 25 ℃; (**b**) 35 ℃; (**c**) 45 ℃; (**d**) 55 ℃.

The experimental results of stress-AE energy-AE cumulative energy under varying temperatures are shown in Fig. 9. At temperatures of 25°C, 35°C, 45°C, and 55°C, the slow-growth stage persisted for 1001 s, 908 s, 669 s, and 474 s respectively. The corresponding stresses at the first energy leap point were 42.5 MPa, 41.3 MPa, 36.5 MPa, and 30.5 MPa respectively, demonstrating an inverse correlation with increasing temperature. Notably, the frequency of high-amplitude signals increased during this stage. This phenomenon can be attributed to thermal expansion of the coal matrix during temperature elevation, which gradually reduces and closes internal pores and fractures. Consequently, both the compressive resistance and plastic deformation capacity of the coal mass are weakened, resulting in more intense energy release from micro-fracture damage during the accelerated growth stage.

## Numerical simulation and result analysis

Screen Pipe Stabilization Technology, as one of the core techniques for preventing and controlling borehole instability in underground coal mines, achieves dual functions of mechanical support for borehole walls and regulation of seepage channels through its porous tubular structure. Based on the coupling theory of seepage mechanics and rock mass structure, this technology compensates for stress in strength-degraded zones of surrounding rocks through the inherent strength of screen pipes while maintaining gas drainage efficiency via the pore network in pipe walls. Therefore, screen pipe conditions must be incorporated when analyzing the dominant control factors of borehole instability to achieve targeted control of these factors from a comprehensive process perspective.

### Model establishment and parameter setting

**Fig. 10:**
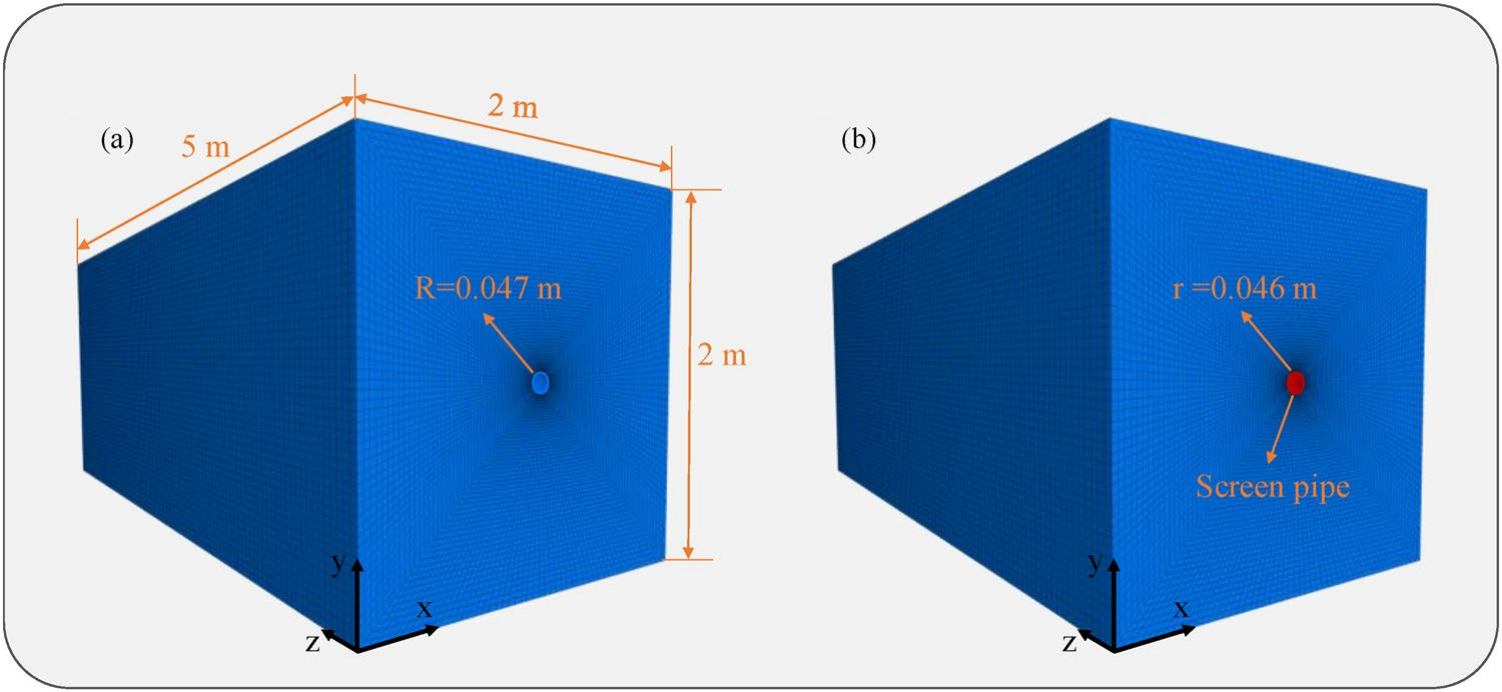
Numerical model.

Using FLAC 3D software with the Mohr–Coulomb model, a 3D numerical model measuring length × width × height = 2 m × 2 m × 5 m was established. The borehole is positioned at the model center with a diameter of 94 mm, as shown in Fig. 10a. A supported borehole model incorporating screen pipes was also developed (Fig. 10b), featuring screen pipes with 94 mm outer diameter and 92 mm inner diameter. An elastic constitutive model guides the screen pipe.

To enable comparative analysis with experimental data, the relevant physical and mechanical parameters of the coal block (at 25℃) are set as shown in Table 3. The simulated confining pressure was set to be 8 MPa, axial pressure to be 12 MPa, and gas pressure to be 1.4 MPa. The screen pipes provide additional confining pressure through their circumferential stiffness. The elastic modulus of the screen pipe was 3 GPa, Poisson’s ratio was 0.3.

**Table 3:** Physical and mechanical parameters.

Bulk modulus (MPa)	Shear modulus (MPa)	Cohesion (MPa)	Tensile strength (MPa)	Friction angle (°)	Density (kg·m^-3^)
1650	600	0.6	0.5	23	1400

### The influence of screen pipes on borehole instability

**Fig. 11:**
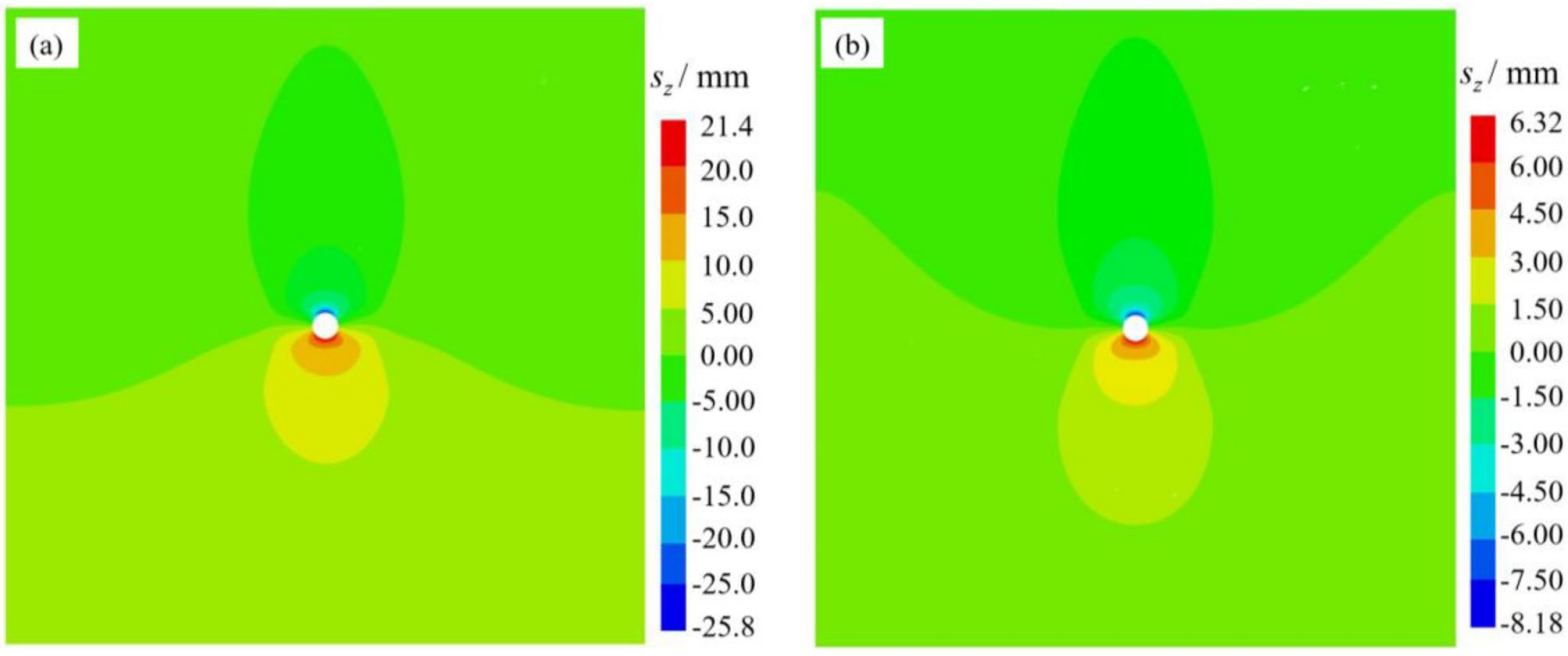
Vertical displacement cloud: (**a**) no screen pipe support; (**b**) screen pipe support.

**Fig. 12:**
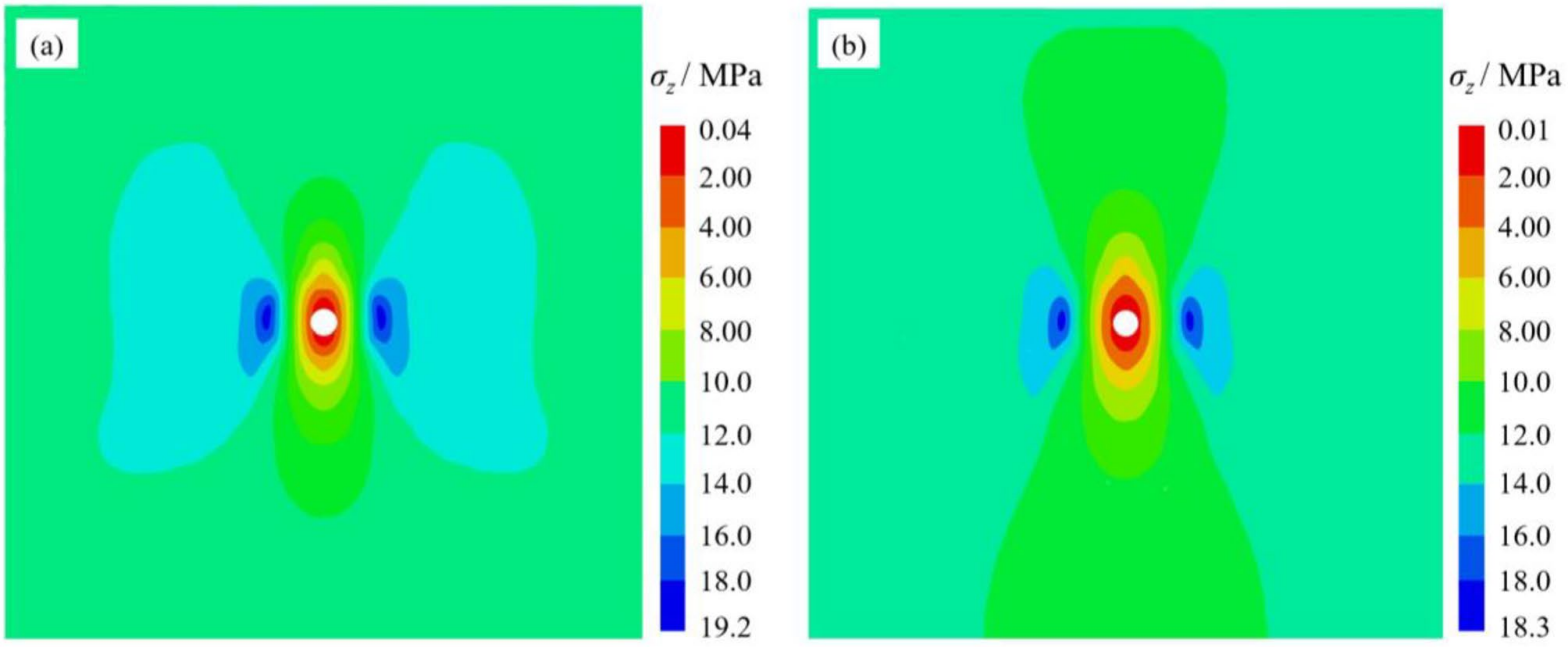
Vertical stress cloud: (**a**) no screen pipe support; (**b**) screen pipe support.

After achieving equilibrium in the model calculation, the distribution results of vertical displacement and vertical stress are shown in Figs. 11 and 12 respectively.

The results demonstrate that screen pipe support significantly enhances the stability of borehole-surrounding rock. In terms of displacement control, the screen pipe support reduces the total vertical displacement of the surrounding rock from 47.2 mm to 14.5 mm, achieving a 69.3% reduction. This constraint effectively minimized deformation and reduced the extent of the loosened zone. In terms of stress optimization, the maximum vertical stress is reduced from 19.2 MPa to 18.3 MPa, and the stress concentration factor is reduced from 1.6 to 1.5. At the same time, the scope of the stress concentration zone is notably narrowed. This shows that the screen pipe support enhances the stability of the borehole through a dual mechanism: on the one hand, it limits the displacement development of the surrounding rock through rigid constraints, and on the other hand, it improves the force state of the surrounding rock through stress redistribution and reduces the degree of stress concentration, which significantly improves the overall stability of the borehole structure.

**Fig. 13:**
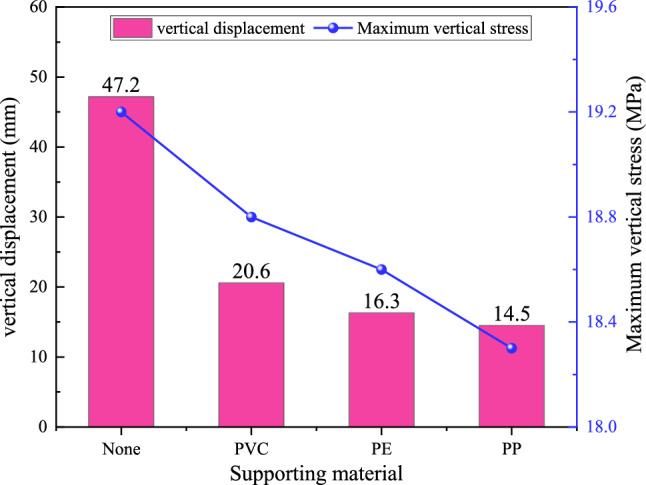
Simulation results with different support materials.

In addition, corresponding numerical simulations were carried out for three commonly used engineering plastics for screen pipes. The basic mechanical parameters of the screen pipes are shown in Table 4, and the statistics of the simulation results are shown in Fig. 13.

**Table 4:** Supporting materials and their mechanical parameters.

Support material	Elastic modulus (GPa)	Poisson 's ratio
Polyvinyl chloride (PVC)	0.7	0.3
Polyethylene (PE)	1.5	0.3
Polypropylene (PP)	3.0	0.3

As shown in Fig. 13, the three screen pipe materials have limited influence on the maximum vertical stress around the borehole. While the PP screen pipe demonstrates the lowest vertical stress, this reduction reaches only 4.7% compared to the unsupported scenario. In contrast, the total vertical displacements decrease by 56.4%, 65.5%, and 69.3% under PVC, PE, and PP screen pipe supports, respectively, compared to the unsupported scenario. The PP screen pipe is also the best displacement constraint under the three support materials. Therefore, combined with the displacement control effect and stress distribution characteristics, the PP screen pipe shows the best support performance.

### Influence of screen pipe support on mechanical properties of coal body

**Fig. 14:**
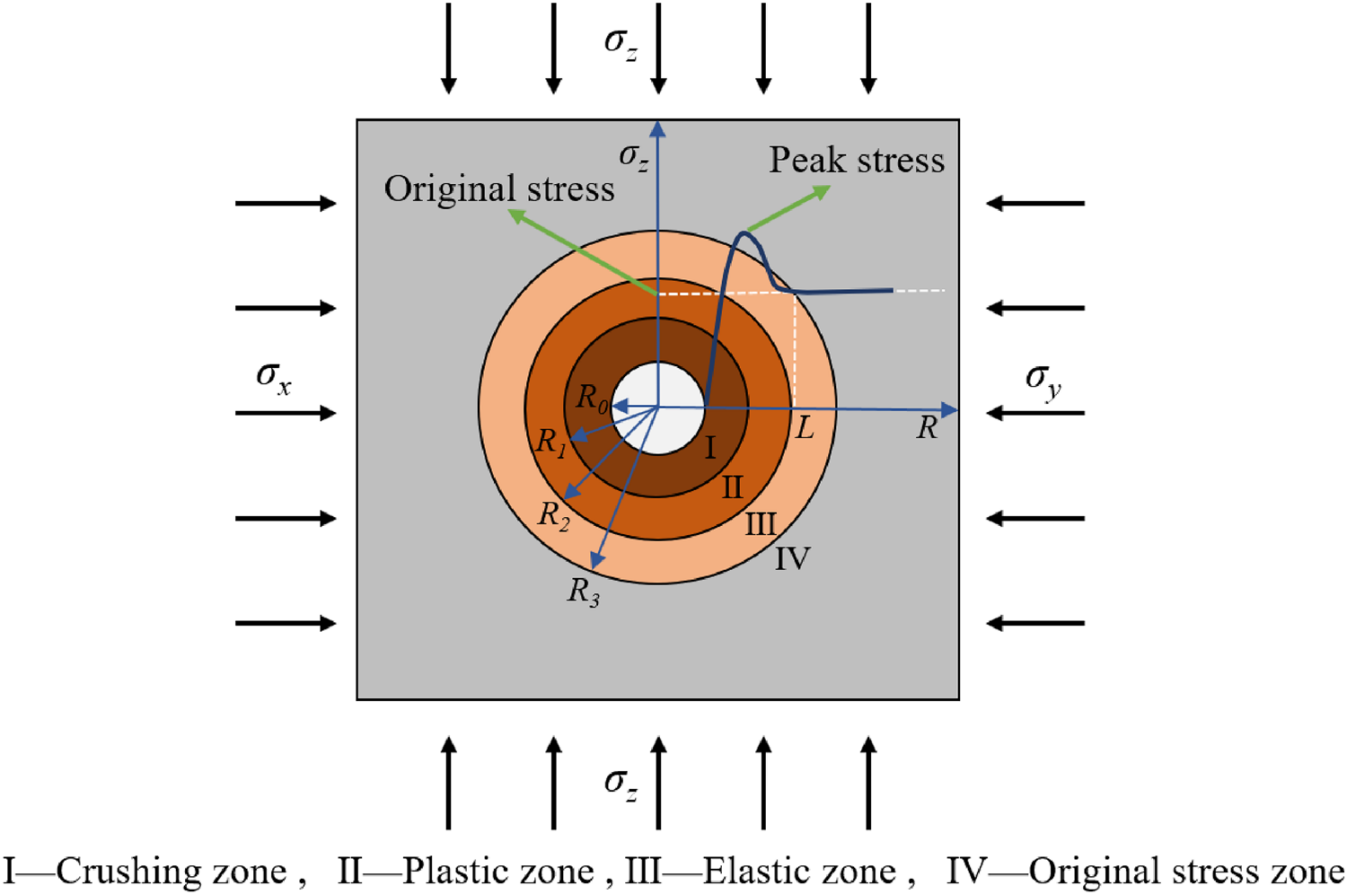
Stress distribution of the borehole surrounding rock.

Under excavation-induced stress redistribution, the abrupt vertical stress reduction at the borehole wall triggers localized stress concentration, initiating the propagation of microfractures within the coal matrix. When stresses surpass the peak strength of the surrounding rock, a plastic zone develops, followed by progressive crushing near the borehole wall under seepage-stress coupling effects. This process ultimately generates a characteristic four-zone stress distribution in the surrounding rock: crushing zone (*R*_*0*_-*R*_*1*_), plastic softening zone (*R*_*1*_-*R*_*2*_), elastic transition zone (*R*_*2*_-*R*_*3*_), protolithic stress zone (> *R*_3_), as illustrated in Fig. 14.

**Fig. 15:**
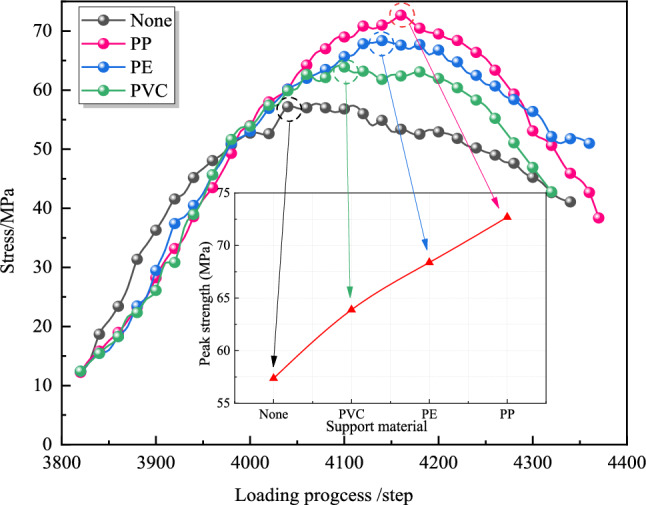
Vertical stress evolution process.

Stress monitoring points positioned at critical interfaces between elastic and protolithic zones across four models captured vertical stress evolution during axial loading (0.0005 m/step) until shear failure. The vertical stress evolution process is shown in Fig. 15.

Results demonstrate significant stress enhancement under screen pipe support, with PP screen pipes achieving a peak stress of 72.5 MPa—27.2% higher than unsupported cases. This improvement stems from three synergistic mechanisms:

1. *Geometric optimization effect of the screen pipe structure* The unique thin-shell curved surface of the screen pipe transforms point loads into surface loads through the geometric continuity of the surface when subjected to external loads. This stress redistribution mechanism controls the local overburden stress within the strength threshold of the coal body, effectively improving the bearing capacity of the 'support material-surrounding rock’ community.
2. *Dynamic energy dissipation mechanism* The low-modulus material absorbs dynamic energy via elastic deformation, reducing stress amplitudes transmitted to the coal body and delaying fracture network propagation. This buffering effect can slow down the fatigue expansion rate of the coal body fracture network, thereby extending the wellbore stabilization period.
3. *Constraint strengthening effect* The composite support system formed by the screen pipe and the surrounding rock of the borehole produces two-way constraints: radial constraints suppress coal dilation, while tangential constraints improve shear resistance.

These findings confirm that screen pipe support critically inhibits borehole destabilization in gas extraction systems, necessitating its inclusion as a key factor in coal seam stability analysis.

### Identification of the dominant controlling factor

Taking confining pressure, gas pressure, temperature, and support material as the main influencing factors with peak stress as the response variable, the Box-Behnken experimental design model^43,44^ was employed as shown in Table 5. Following laboratory experiments and numerical simulations, the experimental results are presented in Table 6.

**Table 5:** Experimental design for four-factor three-level response surface analysis.

Factor		Level		
		− 1	0	1
A	Confining pressure	6	9	12
B	Gas pressure	1.0	1.6	2.2
C	Temperature	25	40	55
D	Support material	None	PE	PP

**Table 6:** Experimental results of response surface analysis.

Number	A	B	C	D	Peak stress	Number	A	B	C	D	Peak stress
1	6	1.0	40	PE	46.5	16	9	2.2	55	PE	53.2
2	6	2.2	40	PE	35.1	17	6	1.6	25	PE	47.4
3	12	1.0	40	PE	80.1	18	6	1.6	55	PE	36.3
4	12	2.2	40	PE	67.2	19	12	1.6	25	PE	78.1
5	9	1.6	25	None	62.4	20	12	1.6	55	PE	69.7
6	9	1.6	25	PP	74.2	21	9	1.0	40	None	65.3
7	9	1.6	55	None	53.5	22	9	1.0	40	PP	72.2
8	9	1.6	55	PP	40.6	23	9	2.2	40	None	43.4
9	6	1.6	40	None	35.6	24	9	2.2	40	PP	58.6
10	6	1.6	40	PP	43.6	25	9	1.6	40	PE	67.5
11	12	1.6	40	None	70.2	26	9	1.6	40	PE	68.2
12	12	1.6	40	PP	74.8	27	9	1.6	40	PE	69.7
13	9	1.0	25	PE	71.1	28	9	1.6	40	PE	68.5
14	9	1.0	55	PE	65.9	29	9	1.6	40	PE	67.2
15	9	2.2	25	PE	63.3						

By performing multiple quadratic regression on the experimental results, the response surface equation is obtained as follows:

1 $$\begin{aligned} y = & - 81.801 + 20.161x_{1} + 14.204x_{2} + 1.235x_{3} + 29.217x_{4} - 0.278x_{1} x_{2} \\ & + 0.011x_{1} x_{3} - 0.333x_{1} x_{4} - 0.111x_{2} x_{3} + 3.333x_{2} x_{4} - 0.417x_{3} x_{4} \\ & - 0.799x_{1}^{2} - 6.782x_{2}^{2} - 0.015x_{3}^{2} - 6.067x_{4}^{2} \\ \end{aligned}$$

where, *y* is the peak stress, MPa; *x*_*1*_ is the confining pressure, MPa; *x*_*2*_ is the gas pressure, MPa; *x*_*3*_ is the temperature, °C; and *x*_*4*_ is the support material.

**Fig. 16:**
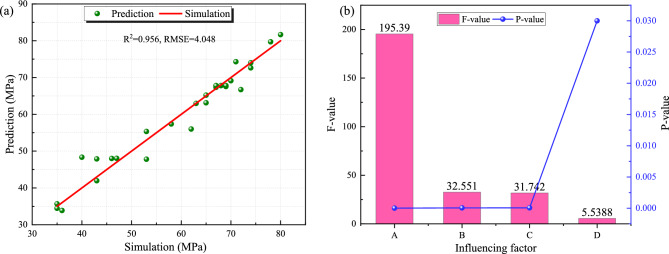
Response surface method analysis results: (**a**) comparison of simulated and predicted values of peak stress in different parameters; (**b**) F and P values for main influencing factors.

The p-value of the model is less than 0.0001, so the regression model demonstrates exceptional statistical significance^45^. Comparison of simulated and predicted values of peak stress in different parameters is shown in Fig. 16a. The F and P values for confining pressure, gas pressure, temperature and support material are shown in Fig. 16b.

Key findings reveal: (1) A strong model validity with predicted values tightly clustered along the y = x line (R^2^ = 0.956), confirming robust predictive capability for engineering applications. (2) Significant parameter correlations through hypothesis testing, where confining pressure (P = 0.00001), gas pressure (P = 0.00005), temperature (P = 0.00006), and support material (P = 0.03) all exhibit P-values below the 0.05 significance threshold. Notably, the screen pipe parameter displays a significance level approximately three orders of magnitude higher than other factors. The screen pipe selection constitutes the primary engineering intervention point for stability enhancement. (3) The F-value hierarchy (confining pressure > gas pressure > temperature > support material) quantitatively verifies that confining pressure dominates borehole instability.

## Conclusion

This study systematically investigates the stability mechanisms of gas extraction boreholes in coal seams through integrated physical experiments and numerical simulations, employing response surface methodology to identify critical control factors. The specific conclusions are as follows:

1. Confining pressure exerts dominant mechanical enhancements, increasing the peak strength by 76.1% and the elastic modulus by 28.1%. Conversely, elevated gas pressure induces strength degradation, which reduced the peak strength by 22.6% and the elastic modulus by 32.0%. Increasing temperature degrades peak strength by 37.1% and the elastic modulus follows a non-linear trend with a net increase of 11.1%.
2. According to the different time of the first energy leap point, the AE energy evolution shows the characteristics of ‘slow-accelerating’ two stages, and the stress at the leap point is positively correlated with the confining pressure, and negatively correlated with the gas pressure and temperature.
3. The screen pipe has obvious inhibiting effect on the instability damage of gas drainage borehole, the PP screen pipe is better than PVC and PE screen pipes, and can reduce the vertical displacement by 69.3%. Under axial loading, the bearing capacity of PP screen pipes and surrounding rock community is increased by 27.2%.
4. Confining pressure is the dominant controlling factor for the instability of gas drainage borehole in coal seam. Elevated gas pressure and thermal deterioration of temperature are accelerators of damage accumulation and energy storage structure collapse, respectively. Screen pipes function as deformation inhibitors for boreholes.

## Data Availability

The datasets used and analysed during the current study available from the corresponding author on reasonable request.

## References

[1] An, Y. et al. Numerical simulation of deep coalbed methane development based on embedded discrete fracture model: A triple medium flow approach. *Energy Sci. Eng.* **2025**, 1–18 (2025).

[2] Lin, D. Y. et al. Characteristics of coalbed methane resources of china. *Acta Geol. Sin.-English Ed.* **74**(3), 706–710 (2000).

[3] So, X. B., Zhang, L. P. & Zhang, R. L. The abnormal pressure regime of the Pennsylvanian no. 8 coalbed methane reservoir in Liulin-Wupu district, Eastern Ordos Basin, China. *Int. J. Coal Geol.* **53**(4), 227–239 (2003).

[4] He, S. et al. Borehole protection technology of screen pipes for gas drainage boreholes in soft coal seams. *Energies* **15**(15), 5657 (2022).

[5] He, S. et al. Failure mechanism of methane drainage borehole in soft coal seams: Insights from simulation, theoretical analysis and in-borehole imaging. *Process Saf. Environ. Prot.* **168**, 410–421 (2022).

[6] Liu, C. et al. Failure analysis of borehole liners in soft coal seam for gas drainage. *Eng. Fail. Anal.* **42**, 274–283 (2014).

[7] Wang, K. & Du, F. Coal-gas compound dynamic disasters in China: A review. *Process Saf. Environ. Prot.* **133**, 1–17 (2020).

[8] Karacan, C. Ö., Ruiz, F. A., Cotè, M. & Phipps, S. Coal mine methane: A review of capture and utilization practices with benefits to mining safety and to greenhouse gas reduction. *Int. J. Coal Geol.* **86**(2–3), 121–156 (2011).

[9] Wei, Z. et al. Effect of acidification on microscopic properties and pore structure of coal. *Fuel* **343**, 127834 (2023).

[10] Zhang, H. et al. A novel in-seam borehole discontinuous hydraulic flushing technology in the driving face of soft coal seams: Enhanced gas extraction mechanism and field application. *Rock Mech. Rock Eng.* **55**(2), 885–907 (2022).

[11] Guo, H., Todhunter, C., Qu, Q. & Qin, Z. Longwall horizontal gas drainage through goaf pressure control. *Int. J. Coal Geol.* **150**, 276–286 (2015).

[12] Liu, J. et al. Study on effective extraction radius of directional long borehole and analysis of the influence mechanism. *ACS Omega* **8**(2), 2344–2356 (2023). 36687070 10.1021/acsomega.2c06749 PMC9850474

[13] Hu, T. et al. Prediction and design of grouting parameters for long and large-diameter postgrouted drilled shafts. *Int. J. Geomech.* **24**(2), 4930 (2024).

[14] Zhang, R. et al. Fractal characteristics of acoustic emission of gas-bearing coal subjected to true triaxial loading. *Measurement* **169**, 108349 (2021).

[15] Zhang, W. et al. A new monitoring-while-drilling method of large diameter drilling in underground coal mine and their application. *Measurement* **173**, 108840 (2021).

[16] Zhai, C. et al. novel active prevention technology for borehole instability under the influence of mining activities. *J. Nat. Gas Sci. Eng.* **27**, 1585–1596 (2015).

[17] Zingg, S. & Anagnostou, G. Effects of the hydraulic capacity of advance drainage boreholes on tunnel face stability. *Tunn. Undergr. Space Technol.* **71**, 518–530 (2018).

[18] Ma, J. & Zhao, G. Borehole stability analysis in fractured porous media associated with elastoplastic damage response. *Int. J. Geomech.* **18**(5), 90–96 (2018).

[19] Saliya, K. et al. Thermo-hydro-mechanical modeling with langmuir’s adsorption isotherm of the co2 injection in coal. *Int. J. Numer. Anal. Meth. Geomech.* **39**(6), 594–617 (2015).

[20] Zhao, Y., Lin, B. & Liu, T. Thermo-hydro-mechanical couplings controlling gas migration in heterogeneous and elastically-deformed coal. *Comput. Geotech.* **123**, 103570 (2020).

[21] Niu, Y. et al. A new method of monitoring the stability of boreholes for methane drainage from coal seams. *Measurement* **154**, 107521 (2020).

[22] Zhang, X., Wang, W. & Yang, M. Study on deformation and destabilization characteristics and modes of drainage borehole. *Energy Sources Part A* **42**(19), 2448–2459 (2020).

[23] Wang, D. et al. Non-linear response of acoustic emission and electric potential during creep failure of coal under stepwise increasing loads: Insights from multifractal theory. *Nat. Resour. Res.* **33**(5), 2113–2133 (2024).

[24] Yu, Y. et al. Deformation mechanism and damage energy evolution of coal body under different gas pressures based on the energy principle. *Sci. Rep.* **15**(1), 3001 (2025). 39849043 10.1038/s41598-025-87373-1 PMC11757720

[25] Liu, S. et al. Experimental study on the microstructure evolution laws in coal seam affected by temperature impact. *Rock Mech. Rock Eng.* **53**(3), 1359–1374 (2020).

[26] Liu, T. et al. Evolution of coal fractures and its influence on permeability during progressive failure based on in situ ct scanning. *Energy Fuels* **38**(15), 14119–14135 (2024).

[27] Wang, D. et al. Quantitative analysis of fracture dynamic evolution in coal subjected to uniaxial and triaxial compression loads based on industrial CT and fractal theory. *J. Petrol. Sci. Eng.* **196**, 108051 (2021).

[28] Shi, J. et al. Experimental study on permeability evolution of bituminous coal under high temperature and volumetric stress. *Rock Mech. Rock Eng.* **56**(7), 5223–5239 (2023).

[29] Shu, C. et al. Temperature variations of coal in the heading face measured using a thermo-hydro-mechanical model considering desorptional heat. *Appl. Therm. Eng.* **181**, 115969 (2020).

[30] Fan, C. et al. Modelling and optimization of enhanced coalbed methane recovery using co2/n2 mixtures. *Fuel* **253**, 1114–1129 (2019).

[31] Fan, C. et al. Thermo-hydro-mechanical-chemical couplings controlling ch4 production and co2 sequestration in enhanced coalbed methane recovery. *Energy* **173**, 1054–1077 (2019).

[32] Zha, W. et al. Modeling methane adsorption distance using carbon nanotubes and bituminous coal pore models. *Energy Fuels* **38**(2), 948–960 (2024).

[33] Wang, K. et al. Transition of dominated factors in coal seam gas migration: Thermo-hydro-mechanical modeling and analysis. *Int. J. Heat Mass Transf.* **236**(1), 126239 (2025).

[34] Liu, J. et al. Coupled thermo-hydro-mechanical cohesive phase-field model for hydraulic fracturing in deep coal seams. *Appl. Math. Mech.-English Ed.* **46**(4), 663–682 (2025).

[35] Yuan, X. et al. Dynamic gas emission during coal seam drilling under the thermo-hydro-mechanical coupling effect: A theoretical model and numerical simulations. *Gas Sci. Eng.* **131**, 205454 (2024).

[36] Yuan, X. et al. Prediction model and risk assessment of dynamic gas emission during drilling in faulted coal seams. *Phys. Fluids* **36**(11), 113103 (2024).

[37] Gaede, O., Karrech, A. & Regenauer-Lieb, K. Anisotropic damage mechanics as a novel approach to improve pre- and post-failure borehole stability analysis. *Geophys. J. Int.* **193**(3), 1095–1109 (2013).

[38] Zhang, X. et al. Development and application of high-performance drilling hole protection materials. *Sci. Rep.* **15**(1), 11367 (2025). 40175440 10.1038/s41598-025-90076-2 PMC11965417

[39] Zhao, Y. et al. Well completion technology using screen pipe for horizontally- intersected well in soft coal seam. *Procedia Eng.* **73**, 311–317 (2014).

[40] Song, J. et al. Numerical and field investigations of acoustic emission laws of coal fracture under hydro-mechanical coupling loading. *Materials* **15**(19), 6510 (2022). 36233850 10.3390/ma15196510 PMC9572184

[41] Kong, X. et al. Time-varying characteristics of acoustic emission and fractals based on information dimension during structural failure of coal subjected to uniaxial compression. *Measurement* **236**, 115088 (2024).

[42] Ishida, T. et al. Characteristics of carbon dioxide fracturing in comparison to conventional water hydraulic fracturing: Evidence from acoustic emission monitoring of small-scale field experiments. *Int. J. Rock Mech. Min. Sci.* **172**, 105604 (2023).

[43] Hansdah, P., Kumar, S. & Mandre, N. R. Performance optimization of dewatering of coal fine tailings using box-behnken design. *Energy Sources Part A* **40**(1), 75–80 (2018).

[44] Kumar, S., Bhattacharya, S. & Mandre, N. R. Modeling of settling rate of coal fine tailings using 3d response surface methodology. *J. Dispers. Sci. Technol.* **37**(2), 251–257 (2016).

[45] Yadav, A. M., Nikkam, S., Gajbhiye, P. & Tyeb, M. H. Modeling and optimization of coal oil agglomeration using response surface methodology and artificial neural network approaches. *Int. J. Miner. Process.* **163**, 55–63 (2017).

